# Prevalence of neurocognitive dysfunction and associated risk factors among follow-up diabetic type 2 patients in diabetes center of Al-Thawra hospital, Sana^’^a, Yemen

**DOI:** 10.3389/fendo.2026.1873295

**Published:** 2026-07-16

**Authors:** Aisha A. Saber, Ahmed M. Al-Ghani, Abdulrahman A. Humaid

**Affiliations:** 1 1Department of Clinical Biochemistry and Molecular Biology, Faculty of Medicine and Health Sciences, Sana’a University, Sana’a, Yemen; 2 Department of Pharmacy, Faculty of Medicine and Health Sciences, Al-Razi University, Sana’a, Yemen; 3 Department of Microbiology, Faculty of the Sciences, Sana’a University, Sana’a, Yemen; 4 Department of Pharmacy, Faculty of Medical Sciences, University of Modern Sciences, Sana’a, Yemen; 5 Department of Pharmacy, Faculty of Medicine and Health Sciences, Amran University, Amran, Yemen

**Keywords:** neurocognitive dysfunction, prevalence, risk factor, T2DM, Yemen

## Abstract

**Background:**

Diabetes mellitus type 2 is a chronic metabolic disorder which characterized by increased blood glucose that affects different body systems. Increasing blood glucose to high levels cause sorbitol induced blood vessel damage, brain malfunction and degeneration of the nerves that can lead to cognitive dysfunction or dementia. For the best of our knowledge, this is the first study in Yemen, which demonstrate the association between neurocognitive dysfunction and diabetes. This study aimed to determine the prevalence of neurocognitive dysfunction among Yemeni diabetic type2 patients and investigate the relations between these dysfunction, diabetic risk factors, and sociodemographic characteristics.

**Methods:**

The present cross-sectional study was carried out in Diabetes center of Al-Thawra public hospital from November 2024 to January 2025 and the sample size was 400 T2DM patients (aged 35 to 65 years) which calculated by Steven Thompson Equation. Statistical analysis was performed by SPSS program and Mini-Mental State Examination (MMSE) was used for screening neurocognitive dysfunctions (NCDs) as a score varying from zero to 30, and impairment is indicated by a score of 23 or lower.

**Results:**

the result showed that, higher prevalence of (NCDs) 43% among T2DM patients (219 (54.75%) male and 181(45.25%) female). Neurocognitive dysfunction was significantly increased in elderly, low-educated, non-employed and long duration diabetes patients with *P*- value (0.001, 0.001, 0.001, 0.001) respectively. While it was significantly decreased in patients with low BMI and those make regular exercise with (*P*- value = 0.002 and 0.002) respectively.

**Conclusion:**

the study showed a relatively high prevalence of neurocognitive dysfunction among patients with T2DM. In addition to that, neurocognitive dysfunction was more common among older patients, higher BMI, lower education, unemployment, diabetes duration and complications.

## Introduction

According to data published by the World Health Organization, there are more than 400 million people worldwide with diabetes, a disease that represents a significant burden in terms of incidence and mortality rates (1, 2), while, the International Diabetes Federation estimates that there are approximately 589 million adults with diabetes, of which 5.5% are in Yemen (3, 4).

Diabetes mellitus is a group of metabolic disorders characterized by elevated blood glucose levels due to impaired insulin secretion, action, or both. Chronic hyperglycemia is associated with long-term damage to many organs, including the heart, kidneys, nerves, eyes, and blood vessels (5, 6). One of the most newly detected problems of diabetes is the progressive decrease in cognition and mental ability, to be specific, there is a decrease in verbal memory, processing speed and executive functions, Conversely, attention and visuospatial, semantic, and linguistic functions appear to be preserved; however, it has been found that controlling blood glucose levels helps to delay these effects (7–10).

Risk factors that may contribute to the development of cognitive impairment in patients include insulin resistance and obesity (common risk factors for both diabetes and cognitive decline), chronic low-grade inflammation, low or high blood glucose levels, and cardiovascular complications associated with diabetes. Diabetes is known to affect various cognitive abilities, such as memory and processing speed, although executive functions may remain intact (11, 12).

Neurocognitive dysfunction (NCDs) is a broad term that related to mental processes associated with acquiring knowledge, manipulating information, and reasoning. Kept cognitive functioning is integral to maintaining a healthy, active, and independent lifestyle (13). Dementia affects approximately 50 million people worldwide, and this number is expected to rise to 82 million by 2030. Previous reviews have concentrated on the relationship between cognitive decline and personal factors such as lifestyle and health status (14).

Cognitive disorders and dementia have multiple causes and diverse clinical manifestations. Recently, defects in insulin signaling in the brain have been linked to cognitive disorders and dementia. In this context, insulin signaling pathways in the brain regulate learning and memory, and modulate energy metabolism in peripheral tissues (15).

High blood sugar levels are linked to cognitive decline and can impair cognitive function in both the short and long term. They are also associated with problems with working memory, attention, and depression. Large fluctuations in blood sugar levels negatively impact cognitive performance, so better glycemic control is beneficial for maintaining healthy cognitive function. This disease does not cause minor structural changes in blood vessels, but it is associated with alterations in regional cerebral blood flow or osmotic changes in neuronal cell membranes. In contrast, chronic hyperglycemia affects cognitive function through the production of advanced glycation end products (AGEs), the formation of senescent plaques and neurofibrillary tangles, and damage to the brain’s microvascular blood vessels. The reduction in white matter volume has also been associated with a decrease in executive functions and a reduction in information processing (16, 17).

A global systematic review indicated a prevalence of NCDs of 21.2%, but regional studies demonstrate different rates, In Europe and Asia, realized that prevalence of mild NCDs among diabetic patients was 45%, with rates of 82.3% in Europe and 98% in Asia. Likewise, a cross-sectional study in India found NCDs prevalence was 33.73%, in one study and 24% in another. In Malaysia, the prevalence was 46.9%, while in Chile, it was 17.3%. A study in Kenya showed a prevalence of 32%. In Ethiopia, various studies have examined NCDs among diabetic patients, including a recent cross-sectional study in Bahir Dar city referral hospitals, which found a prevalence of 27.6%. A study in Saudi Arabia demonstrated an overall prevalence of 80.3%, with 33.8% classified as severe. In Egypt, the prevalence among diabetic patients was 34%, and in Nigeria, it was 40% (18).

The risk of cognitive impairment in DM patients is 1.2 to 1.5-fold higher than that reported in non-DM individuals (19). Several potential mechanisms promote the occurrence of cognitive impairment in T2DM, including hyperglycemic toxicity, insulin resistance, oxidative stress, accumulation of amyloid-beta peptide and tau hyper-phosphorylation (20). Hypertension, diabetes mellitus, obesity, smoking, hyperlipidemia, and a history of cardiovascular diseases, such as stroke and ischemic heart disease, are strongly associated with neurocognitive disorders. Hypertension is known to affect cognitive functions through structural and functional impairment of the cerebral blood vessels and a direct impact on the functions of the central nervous system through changes in the cerebral renin–angiotensin system (21).

Low socioeconomic position (e.g., limited education and low income), unemployment, and poor childhood socioeconomic conditions are linked to higher risks. Higher levels of oxidative stress markers like derivatives of reactive oxygen metabolites and reduced paraoxonase 1 (PON1) activity correlate with impaired cognition (22).

Several studies have reported that many khat users also smoked cigarettes and these concurrent users display impairments in verbal learning, memory recall and working memory. One explanation is that cathinone has been found to act as a presynaptic release and uptake inhibitor of dopamine leading to depletion of serotonin in brain areas involved in spatial learning and memory (23).

For the best of our knowledge, this is the first study in Yemen, which demonstrate the prevalence and associated risk factors of NCDs among T2DM patients.

## Methodology

**Figure 1:**
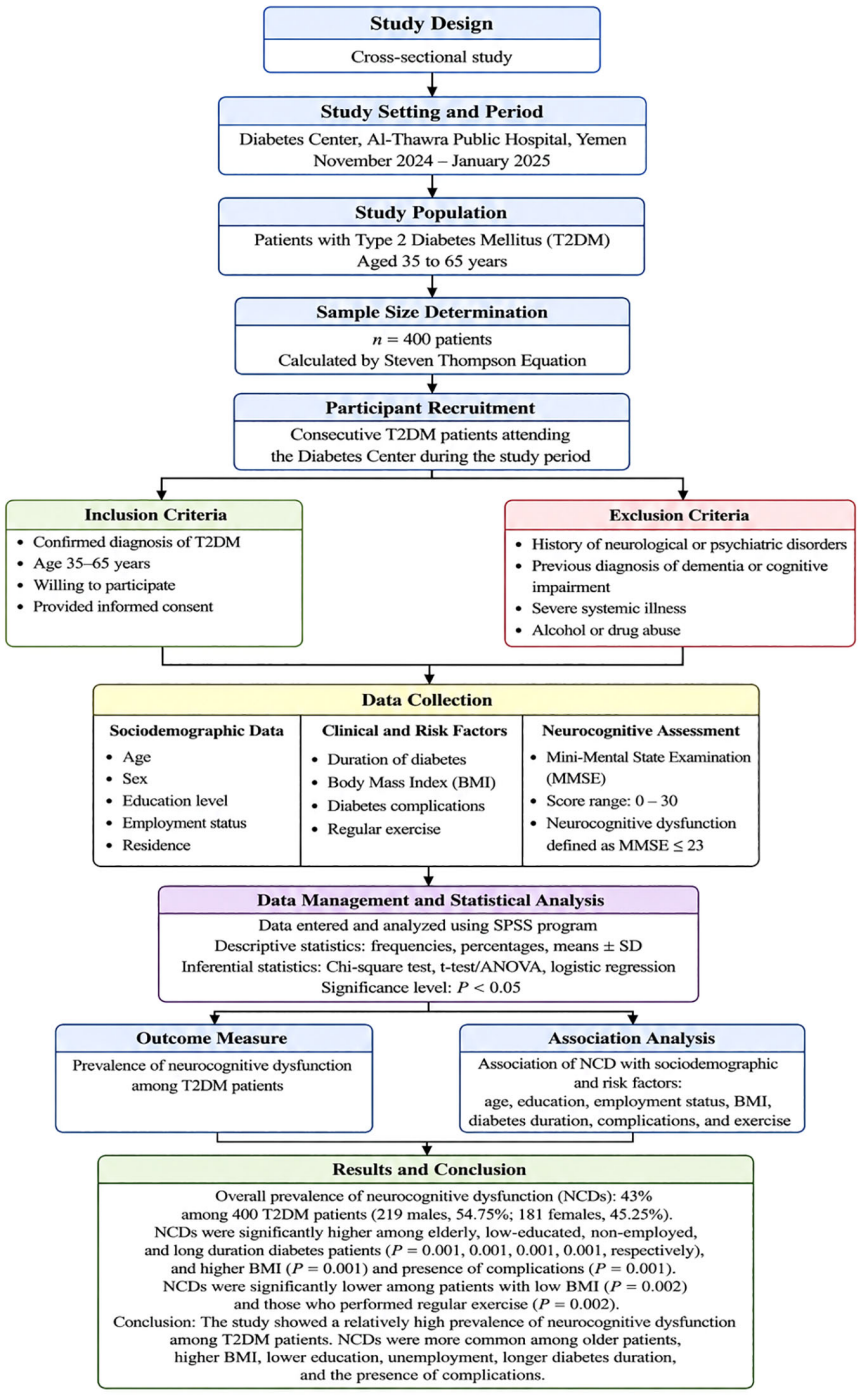
Flowchart outlining the cross-sectional study design investigating neurocognitive dysfunction prevalence in type 2 diabetes patients at a Yemeni hospital, detailing setting, inclusion and exclusion criteria, data collection methods, statistical analysis, outcome measures, association analysis, and summarized results emphasizing higher dysfunction among older, less educated, unemployed, longer-duration diabetes patients, and those with complications or higher BMI.

The study process shows in Methodological flowchart **Figure 1**.

### Study design

Across-sectional study was carried-out at Diabetes Center of AL-Thawra Hospital, Sana’a city, Yemen from November 2024 to January 2025. This design used for the assessment of the relationships between variables at a single time point, participant characteristics and valuable insights concerning prevalence and associations.

### Participants and data collection

Sample size was 400 T2DM Patients, which calculated by Steven Thompson Equation 2012, third, Edition; p: 59–60 according to using 95% Confident interval and number of population size attending the hospital for the last three years was 15098. The study population consisted of adult participants aged 35 years to 65 years. The inclusion criteria needed participants to have a confirmed diagnosis of T2DM. Exclusion criteria included severe mental illness, advanced dementia, or inability to provide informed consent. All participants willing agreed to participate in the study after being well-informed of its objectives and procedures.

A structured questionnaire was designed to gather comprehensive data on participants, medical history, lifestyle habits, and diabetes-related complications.

The structured questionnaire used in this study facilitated standardized and comprehensive collection of demographic and clinical information, thereby improving the uniformity of data acquisition. The Mini-Mental State Examination (MMSE) was employed to assess participants’ neurocognitive functions (24). This widely used tool evaluates orientation, memory, attention, calculation, language, and visuospatial abilities. The MMSE consists of 11 questions, with a maximum score of 30. Scores were categorized as follows:

Normal neurocognitive function: 24–30.
Neurocognitive dysfunction: ≤ 23.

The MMSE is a valid and reliable instrument for screening cognitive impairment, with reported test–retest reliability ranging from 0.80 to 0.95 and high inter-rater reliability. It has been widely used in studies assessing cognitive function among adults with diabetes mellitus (25–27).

Body weight was measured in kilograms using a digital scale, while height was measured in centimeters using a stadiometer. These measurements were used to calculate Body Mass Index (BMI) using the formula:


$$BMI=\frac{Weight\;(kg)}{{\left[Height\;(m)\right]}^{2}}$$

The blood pressure was measured by mercury sphygmomanometer.

Each participant completed the questionnaire, followed by physical measurements and the MMSE assessment. All measurements and assessments were conducted by competent healthcare professionals to ensure accuracy and reliability and all participants were assured of the privacy of their data.

### Data analysis

The Statistical Package for Social Sciences (IBM SPSS) version 26.0 was used to analyze and manage the collected data.

Descriptive statistics were used to describe the prevalence of neurocognitive dysfunctions in diabetic patients, and the relationship between nominal qualitative variables was expressed by the Chi-Square test. The significant differences were indicated if *P-*value was ≤ 0.05.

### Ethical considerations

Subjects were asked to participate in the study prior to the procedure. The investigator was providing the subject with information regarding the research study. All subjects were given informed consent to use their information before the initiation of any study procedures. The consent form would be submitted to the ethical committee of the Faculty of Medicine and Health Sciences, Al-Razi University, for approval.

## Results

### Prevalence of NCDs among T2DM patients

**Table 1** shows that the prevalence of neurocognitive dysfunction among patients with type 2 diabetes mellitus (T2DM) was 171 (43%) out of 400 participants. Among those with neurocognitive dysfunction, 54% were female and 34% were male.

**Table 1:** Prevalence of neurocognitive dysfunction amongT2DM patients.

	Total	Neurocognitive dysfunction n (%)	
		Male	Female
Yes	171 (43%)	74 (33.78%)	97 (53.59%)
No	229 (57%)	145 (66%)	84 (46%)

### The association between participant’s sociodemographic data and NCDs

**Table 2** presents the association between sociodemographic characteristics and neurocognitive dysfunction. Participants aged 56–65 years, those with lower educational attainment and unemployed individuals demonstrated a higher proportion of neurocognitive dysfunction compared with younger participants, individuals with higher education, employed participants, and those who reported engaging in regular exercise. These associations were statistically significant with p-values of 0.001, 0.001, 0.001, and 0.002, respectively.

**Table 2:** The association between sociodemographic data and NCDs.

Variables	n (%)	Non – NCDs n = 229 (57%)	NCDs n=171 (43%)	$x^{2}$	*P value*
Age (years)					
35–45 years	98	85	13	70.971	0.001
46–55 years	148	92	56		
56–65 years	154	52	102		
Education					
Primary	204	55	149	158.636	0.001
Secondary	102	85	17		
Graduated	94	89	5		
Occupation					
Employed	227	151	76	18.428	0.001
Non-Employed	173	78	95		
Smoking					
Smoker	75	44	31	0.076	0.443
Non- smoker	325	185	140		
Diet					
Healthy diet	228	134	94	0.502	0.272
Not-healthy diet	172	95	77		
Khat chewing					
Khat chewer	265	166	99	9.326	0.002
Non-khat chewer	135	63	72		
Exercise					
Regular	268	170	98	12.701	0.002
Never	132	59	73		

$x^{2}$, Chi-square.

*P*, probability value.

### The association between BMI, BP, presence of diabetic complications and duration of diabetes and NCDs

As shown in **Table 3**, neurocognitive dysfunction was significantly less frequent among participants with lower body mass index (BMI) (p = 0.020). In contrast, the presence of diabetic complications and diabetes duration longer than 10 years were significantly associated with higher rates of neurocognitive dysfunction (p = 0.012 and p = 0.001, respectively).

**Table 3:** Effect of BMI, BP, and presence of diabetic complications and duration of diabetes on cognitive function.

Variables	Non – NCDs n = 229	NCDs n =171	*P value*
BMI	26.34 ± 3.63	27.24 ± 4.09	0.020
Systolic BP	120.93 ± 13.55	122.69 ± 15.35	0.228
Diastolic BP	78.75 ± 9.68	80.23 ± 10.38	0.144
Presence of diabetic complications			
Yes	140 (52.8%)	125 (47.2%)	0.012
No	89 (65.9%)	46 (34.1%)	
Duration of diabetes			
Less than 5 years	96 (69.1%)	43 (30.9%)	0.001
5–10 years	56 (56%)	44 (44%)	
More than 10 years	77 (47.8%)	84 (52.2%)	

BMI, Body Mass Index (kg/m²); BP, Blood Pressure (mmHg).

Continuous variables (BMI and BP) were expressed as mean ± standard deviation (SD) and analyzed using an independent sample t-test. Categorical variables such as presence of diabetic complications and duration of diabetes were analyzed using the Chi-square test.

## Discussion

The present study found that the prevalence of neurocognitive dysfunction among Yemeni patients with T2DM was 43%, indicating that cognitive impairment may be relatively common in this population. This finding is broadly consistent with previous international studies. For example, a recent study conducted in India reported a prevalence of 58.3% among patients with T2DM, with diabetes duration ≥7 years identified as a significant risk factor (28). Additionally, a meta-analysis including 30 studies with 10, 469 participants reported an overall prevalence of 44.1**%** for neurocognitive dysfunction among individuals with T2DM (29).

Gray matter atrophy in people with T2DM occurs 20% faster, but less than 14% faster than during normal aging. The neurocognitive impact of T2DM indicates a marked acceleration of normal brain aging (30). Insulin has been proven to play a role in protection of neurocognitive functions. It also has an indirect effect on brain function on peripheral tissues (16). Mechanism of diabetic NCDs in T2DM is a complex but may involves some factors like hyperglycemia, insulin resistance, increases oxidation stress and other inflammatory processes (13). Hyperglycemia induced damage in the neurons of hippocampal and cortical area in brain, which can lead to severe spatial memory and learning dysfunction. Inflammatory cytokines like IL-6 and TNF are increased persistently in patients with T2DM and consequently decreased executive functions (31). Recent studies show that T2DM is linked to widespread metabolic disturbances, including increased oxidative stress, chronic inflammation, and mitochondrial dysfunction, which together play a key role in the progression of neurocognitive decline. These mechanisms are consistently reported as central contributors to diabetes-related complications (32). In addition, growing evidence suggests that plant-derived bioactive compounds may offer neuroprotective benefits by targeting these pathways through their antioxidant and anti-inflammatory properties (32, 33). Overall, these previous study highlight the potential value of natural therapeutic approaches in reducing cognitive impairment associated with metabolic disorders (33).

This study described that, the younger age, educated, employed, and patients having exercise were have low cognitive dysfunction. On the other hand, older persons, low educated, unemployed, were highly associated factors with NCDs and this result was agreed with most previous studies (29, 34, 35). Neurocognitive dysfunction is more common in the elderly because aging is associated with structural and functional brain changes, increased risk of neurodegenerative and vascular diseases, accumulated cellular damage, medical comorbidities, and medication-related effects. These factors together make cognitive impairment and dementia significantly more prevalent in older populations (36).

The individuals with higher education showed lower NCDs risks, emphazing education as a crucial factor in cognitive health and is a more critical determinant of overall cognitive flexibility (37). The association between low educational attainment and neurocognitive dysfunction may be explained by reduced cognitive reserve, decreased engagement in cognitively stimulating activities across the lifespan, and greater exposure to socioeconomic and health-related risk factors that increase vulnerability to cognitive impairment and dementia (38). Employment provides cognitive engagement, social interaction, routine, purpose, and income. These factors can help support cognitive functioning. Conversely, cognitive impairment can reduce employability. Because each can influence the other, studies frequently observe a strong association between unemployment and poorer cognitive performance (39).

Khat contains stimulants mainly cathinone and cathine, which act on dopamine, norepinephrine, and serotonin systems. These neurotransmitters are heavily involved in attention, memory, mood, and executive function (40).

Cognitive impairment among khat users explained by the combination of stimulation, sleep disruption and long-term neurochemical stress which can gradually reduce cognitive performance, especially with heavy or prolonged use (23). Previous study described that, users Khat individuals demonstrated deficits associated with learning compared to non-users, while users were found to have significantly lower scores on the Serial Digit Learning test. They found also significant deficits in several cognitive domains, including learning, motor speed/coordination, set-shifting/response inhibition functions, cognitive flexibility, short-term working memory and conflict resolution is associated with long term khat use (23). The present study found a statistically significant association between khat use and neurocognitive dysfunction (χ² = 9.326, p = 0.002). Notably, a greater proportion of neurocognitive dysfunction was observed among non-khat users compared with khat users. This unexpected finding may be explained by several factors. First, confounding variables such as age, education level, neurological conditions, and socioeconomic status may have influenced cognitive outcomes. If khat users are predominantly younger or more socially active individuals, this may partially reduce the likelihood of observable cognitive decline in a cross-sectional study design. Second, selection bias may have affected the composition of the groups, as khat users and non-users may differ systematically in their baseline characteristics. Third, reverse causation may also contribute, whereby individuals with cognitive impairment are less likely to use khat.

Studies have been extensively focused on neural plasticity, the direct target of exercise in the brain. In addition, mitochondrial stability and energy metabolism are essential for brain status and the organ-brain axis responds to exercise and induces release of cytokines related to cognition (41).

The results of this research showed that a high body mass index, the presence of diabetes-related complications, and a duration of diabetes exceeding 10 years are among the factors that associated with a significant increase in the incidence of NCDs. An adipose tissue increases the risk of vascular disorders by secreting biological active hormonal compound. In addition to that, obesity can lead to the accumulation of brain lesions due to vascular and metabolic abnormalities, which can increase the risk NCDs (42). Obesity is associated with numerous harmful pathological changes, including insulin resistance, gut microbiota imbalance (dysbiosis), oxidative stress, inflammation activation, and systemic inflammation, all of which can contribute to neuroinflammation and subsequent brain injury (43). Insulin signaling pathways in the brain regulate learning and memory, and modulate peripheral energy metabolism consequently long duration of diabetes and impaired insulin signaling in the brain have been associated with cognitive Impairment and dementia (15).

## Conclusion

This study found that neurocognitive dysfunction was present in 43% of patients with type 2 diabetes mellitus in the study population. Older age, lower educational level, unemployment, higher BMI, longer duration of diabetes, and the presence of diabetic complications were significantly associated with higher rates of neurocognitive dysfunction.

While these findings suggest that several demographic and clinical factors may be associated with cognitive impairment among patients with T2DM, the cross-sectional nature of the study limits causal interpretation. Further longitudinal and multicenter studies are required to better understand the mechanisms and risk factors contributing to neurocognitive dysfunction in diabetic populations.

## Future research recommendations

*Prospective longitudinal designs are also needed to better understand the direction of the relationship between diabetes and cognitive decline, and to clarify whether factors such as chronic hyperglycemia or vascular complications contribute most significantly to cognitive impairment.

*In addition, incorporating neuroimaging techniques and biological markers such as HbA1c fluctuations, inflammatory indicators, and measures of insulin resistance may help explain the underlying mechanisms involved.

*It is also important that future research carefully adjusts for potential confounding variables, including aging, educational background, hypertension, depression, and cardiovascular comorbidities, all of which can independently influence cognitive function.

*We recommend that future studies incorporate a more comprehensive range of clinical and metabolic variables.

*Moreover, there is a need for more evidence from low- and middle-income countries to improve the global representativeness of findings, as most existing studies have been conducted in high-income settings.

## Limitation of this study

First, cross sectional study makes it hard to know whether the effect is due to the diabetes or other factors. Other confounding factors such as age, education level or lifestyle may have influenced the results. Furthermore, presence of different tools for assessment of cognitive functions leading to inconsistent finding across different studies. Each tool has different sensitivity and specificity. Results may vary according to which tool is used. Patient above 65 years were excluded from the study due to difficulty in assessing cognitive functions by screening tools only and they required clinical evaluation and sometimes imaging test (CT or MRI) and this require financial support (budget limitations restricted the depth of assessment). The result cannot be generalized to elderly since cognitive impairment is more common in older populations, excluding them may limit the clinical significance of the finding. Finally difficult reach to the patient diagnostic or therapeutic records (like type of treatment or glycemic control history and other variables) at the time of study, make us exclude it and could not involve this parameter in the study.

## Data Availability

The raw data supporting the conclusions of this article will be made available by the authors, without undue reservation.
